# Regular Khat (*Catha edulis*) chewing is associated with elevated diastolic blood pressure among adults in Butajira, Ethiopia: A comparative study

**DOI:** 10.1186/1471-2458-10-390

**Published:** 2010-07-02

**Authors:** Workineh Getahun, Teferi Gedif, Fikru Tesfaye

**Affiliations:** 1 School of Pharmacy, Addis Ababa University, Addis Ababa, Ethiopia; 2 School of Public Health, Addis Ababa University, Addis Ababa, Ethiopia

## Abstract

**Background:**

Fresh leaves and buds of the Khat plant (*Catha edulis*) contain Cathinone, an amphetamine like alkaloid responsible for its pharmacological action. Chewing of Khat has been associated with a transient rise in blood pressure and heart rate in experimental studies. Few studies examined the effect of regular or frequent Khat chewing on blood pressure at the population level. This study was conducted to examine the association of regular Khat chewing with blood pressure among adults.

**Methods:**

We compared systolic and diastolic blood pressure of adults 35-65 years of age who reported regular chewing of Khat during the preceding five years to those who never chewed Khat during the same period. Study participants were recruited from purposively selected urban and rural villages of Butajira District in Ethiopia. The comparative groups, chewers (334) and non-chewers (330), were identified from among the general population through a house-to-house visit using a screening questionnaire. They were frequency-matched for sex and age within a five-year range. Data were collected through structured interviews and physical measurements including blood pressure, weight and height.

**Results:**

The prevalence of hypertension was significantly higher among Khat chewers (13.4%) than non-chewers (10.7%), odds ratio (OR) = 1.66 (95% confidence interval (CI) 1.05, 3.13). A considerably high proportion of chewers (29.9%) than non-chewers (20.6%) had sub-optimal diastolic blood pressure (> 80 mmHg). The mean (sd) diastolic blood pressure was significantly higher among Khat chewers [75.0 (11.6)] than non-chewers [72.9 (11.7)], P < 0.05. Similarly, Khat chewers had significantly higher mean (sd) heart rate [76.3 (11.5)] than non-chewers [73.9 (12.6)], P < 0.05. There was no significant difference in mean systolic blood pressure between the two groups.

**Conclusion:**

Regular chewing of Khat is associated with elevated mean diastolic blood pressure, which is consistent with the peripheral vasoconstrictor effect of Cathinone. Regular Khat chewing may have sustained effects on the cardiovascular system that can contribute to elevated blood pressure at the population level.

## Background

Hypertension is a growing public health problem, with remarkable contribution to cardiovascular diseases (CVD) morbidity. Worldwide, an estimated 1 billion individuals have hypertension, and approximately 7.1 million deaths per year are attributable to hypertension [1]. It is estimated that more than 20 million people are affected in the African Region, where prevalence ranges from 25 percent to 35 percent in adults aged 25 to 64 years [2]. In Ethiopia, hypertension accounted for 1.4 percent of all deaths reported to Federal Ministry of Health of Ethiopia (FMOH) in 2000/01, making it the 7th leading cause of death in the country for the year [3].

Elevated blood pressure has many risk factors that are of behavioral, dietary or genetic origin. Among the main modifiable risk factors of hypertension are overweight and obesity, cigarette smoking, physical inactivity, unhealthy diet, stress, dietary salt intake, and alcohol use [2,4]. Regular and repeated intake of Khat has recently been reported to be associated with increased risk of acute myocardial infarction [5] and high blood pressure [6].

Khat is found in the flowering evergreen tree or large shrub of Celastracea family. It consists of whole fresh leaves and buds of a plant known as *Catha edulis*. It is indigenous to Ethiopia, Kenya, and Yemen [7]. More than 20 different compounds including, Cathinone/amino propiophenone/, Cathine/nor pseudoephedrine/and nor ephedrine have been isolated from Khat [8]. Cathinone, which is the main active ingredient in Khat leaves, is responsible for the pharmacological properties of Khat [9-11].

The use of Khat is an established cultural tradition for many social situations in the areas of primary cultivation, East Africa and the Arabian Peninsula. Several million people may be chewing Khat worldwide, with an estimated 10 million people chewing Khat leaf daily [12]. The prevalence of Khat chewing in Butajira district was reported to be 70 percent among males and 37 percent among females [13].

Khat typically is ingested while chewing the leaves. After ingesting Khat, the chewer experiences an immediate increase in blood pressure and heart rate [12,14]. Various reasons have been given for chewing Khat. Most chewers used Khat to gain good level of concentration for prayer [15]. Some chewers reported that Khat intake results in increased energy levels and alertness, enhances imaginative ability and the capacity to associate ideas, and improves the ability to communicate [15-17].

Studies on the effect of substance abuse and their health effects are scarce despite the ever-growing rate of substance use behaviors [18]. Some studies, however, indicated that hypertension is among the health consequences of Khat chewing [5,6,8,19,20]. A recent study from an urban population in Ethiopia reported an isolated increase in mean diastolic blood pressure among adults who chewed Khat regularly [6]. The present study examines the association of Khat chewing with systolic and diastolic blood pressure through comparison between Khat chewers and non-chewers in a semi-urban population in central Ethiopia, where the plant is produced and chewed in large quantities.

## Methods

This study was conducted in Meskan District of Gurage Zone, Ethiopia. Meskan District is located 130 km to the south of Addis Ababa. The district population was estimated at 260,000 in 2005, of which 87% live in rural areas. The district covers an area of 797 km^2^. The topography ranges between dry lowlands at altitudes around 1,500 m (tropical climate) to cool mountainous areas of up to 3,500 m above mean sea level (temperate climate). Farming is the main occupation in rural areas while petty trading is common in the town [21]. Khat is cultivated abundantly in the midland and highland parts of the district and chewed widely by both the urban and rural communities [15].

A demographic surveillance system, the Butajira Rural Health Program (BRHP), has been operational in the area since 1987 [21]. The BRHP, implements a continuous demographic surveillance in one urban and nine rural kebeles (smallest administrative units) in the district, which were selected by means of probability proportionate to size (PPS) sampling method. The current study kebeles were drawn from among the ten kebeles that constitute the BRHP [21]. Four out of the ten kebeles (one urban and three rural) were selected purposively, to cover the agro-ecological diversity of the district population (urban, rural lowland, rural midland, and rural highland), which may also reflect the variations in Khat cultivation and use behaviors. In each of the four kebeles, the first household was selected randomly from the list of households maintained by the BRHP data base, after sorting the list of house numbers in a random order using the SPSS version 13 software. Subsequent households were selected on the basis of their proximity to the first and to one another, as commonly applied in the cluster sampling method.

The selection of kebeles or households was not intended to generate a representative sample of the district population. As the primary purpose of the study was the comparison of blood pressure between Khat chewers and non-chewers, efforts were made to ensure that the two groups were selected using the same procedure.

The sample size was estimated using the general formula for comparison of two means [22]. The mean blood pressure and standard deviations of the source population measured in a previous study [23] were taken as a reference for calculating the sample size. The calculated sample size was 660 (including 10% added for non-response rate), consisting of equal proportion of Khat chewers and non-chewers. Of these, 45% was assigned to the urban sample in order to enhance feasibility of data collection.

Once a household was selected, all individuals residing in the household that fulfilled the criteria as either Khat chewer or non chewer were enrolled into the study, until the number of participants assigned to each of the four kebeles (300 to urban, and 100 to each of the rural lowland, midland and highland kebeles, which was further equally divided between Khat chewers and non-chewers) was attained. In the event that there was no eligible individual in the selected household, the next closest household was visited.

Adults between 35 and 65 years of age who chewed Khat for four or more days in a week during the past five years (chewers) or those who never chewed Khat during the past five years (non-chewers), were included in the two groups. Adults younger than 35 year of age were excluded from the study as elevated blood pressure is less common in this age group [23,24], and since focusing on the age group with higher risk of elevated blood pressure yields better in the field data collection process, while serving the purpose of comparison between Khat chewers and non-chewers. Pregnant women were excluded from the study due to the possible confounding effect of pregnancy on the weight and blood pressure.

A semi-structured questionnaire, modified from the World Health Organization instrument for stepwise surveillance (WHO STEPS) of chronic disease risk factors [25,26] was used to collect data. Height was measured and recorded to the nearest 0.5 cm using a wooden measuring board manufactured locally. Weight was measured and recorded to the nearest 100 gm using an ordinary bathroom scale, and waist and hip circumference were measured using a non-elastic tape meter. Blood pressure (BP) was measured using the Omron *M4 *digital blood pressure measuring device.

Twelve data collectors, six male and six female, who have experience in data collection and measurements were recruited and trained on the administration of survey instruments using training manual, developed for the survey based on the WHO training and practical guide [24,25].

Physical measurements of height, weight, hip, and waist circumferences and BP were carried out after the socio-demographic questions have been completed. The interview and physical measurements were conducted during the day, between 8:30 am and 6:30 pm, independent from the time of Khat chewing. Before BP measurement, each study participant was advised to sit and take rest for at least five minutes. Three consecutive measurements were taken on the left arm at 3-5 minutes interval. Average of the second and third measurements was used to describe the mean systolic blood pressure (SBP) and mean diastolic blood pressure (DBP), and heart rate (HR) of study participants as recommended in the WHO STEPS method [25,26].

Data were entered and analyzed using EPI-Info version 6.04d and Statistical Package for Social Sciences (SPSS) version 13.0 software. Proportions and percentages were utilized to describe some characteristics of the study participants. Multiple linear regression analysis was performed in order to identify determinants of DBP and to estimate their corresponding coefficients of variability. Odds ratio (OR) was used to measure association of dependent and independent variables where 95% confidence interval (CI) and P-value were utilized to determine statistical significance.

Ethical clearance for the study was obtained from the Ethics Review Committee of the School of Pharmacy, Addis Ababa University (AAU). Informed and written consent was obtained from each study participant. The survey did not involve any invasive procedure which could cause harm to the study participant or to the community. Individuals with high blood pressure identified during the survey were advised to seek BP measurement service at the local health facilities.

## Results

A total of 667 adults who met the eligibility criteria were interviewed, of which 664 participants have completed both the interview and measurements making the response rate of 99.5%. Among the 664 study participants, 50.3% were Khat chewers and 49.7% were non-chewers. Of the Khat chewers, 53.0% were males, 55.4% were rural residents, 99.4% were married, and 92.5% were Moslem. While the two groups were comparable in most socio demographic characteristics, the non-chewers were less likely to be Moslems (41.5% vs. 92.5%), odds ratio (OR) = 0.07(95% CI (0.04, 0.11). There was no significant difference in mean (sd) age of chewers (43.67) and non-chewers (43.51) (Table 1). Of the non-chewers, 25.2% had chewed Khat before, but not in the past five years.

**Table 1:** Comparison of socio-demographic characteristics of Khat chewers and non-chewers in Butajira, Ethiopia.

Characteristics	Chewers (n = 334)		Non -chewers (n = 330)		OR (95% C I)	
			
	Freq	(Percent)	Freq	(Percent)		
**Sex**						

Male	177	(53.0)	171	(51.8)	1.00	

Female	157	(47.0)	159	(48.2)	0.96	(0.60, 1.54)
**Residence**						

Rural	185	(55.4)	177	(53.6)	1.00	

Urban	149	(44.6)	153	(46.4)	0.93	(0.69,1.27)

**Religion**						

Muslim	309	(92.5)	137	(41.5)	1.00	

Christian	25	(7.5)	193	(58.5)	0.07	(0.04,0.11)

**Ethnic group**						

Meskan	184	(55.1)	128	(38.8)	1.00	

Others	150	(44.9)	202	(61.2)	0.52	(0.38,0.70)

**Educational Status**						

Unable to read or write	192	(57.5)	182	(55.2)	1.00	

Only read and write	61	(18.3)	63	(19.1)	0.92	(0.61,1.38)

Attended formal schooling	81	(24.2)	85	(25.7)	0.90	(0.63,1.30)

**Main Occupation**						

Farmer	138	(41.3)	132	(40.0)	1.00	

Housewife	78	(23.4)	76	(23.0)	0.98	(0.66,1.46)

Merchant	66	(19.8)	68	(20.6)	0.93	(0.61,1.41)

Others	52	(15.5)	54	(16.4)	0.92	(0.59,1.44)

**Mean age (sd)**	43.6	(7.68)	43.5	(7.78)		P > 0.05

sd = standard deviation;

Majority, 237 (70.9%), of chewers chew Khat four days a week. The median duration (years) of chewing was 20 years. The mean (sd) duration of chewing in a day was 2.4 (± 1.2) hours. About half (51.8%) of the Khat chewers chewed Khat for a duration of three hours at a time.

The reported reasons for chewing Khat include religious prayer, to pass time, to accompany or socialize with family members, and to get more concentration at work. Only 30 (9.0%) chewers reported that they might be addicted to Khat, while another 45% claim that they might have some habit of chewing, but no addiction. The remaining 44.4% believed that they have neither addiction nor habituation.

The proportion of smokers was significantly (P < 0.05) higher among Khat chewers (10.8%) than non-chewers (4.8%). The mean (sd) duration of smoking was 16.2 (± 10.5) years; where chewers smoked for mean (sd) of 15.2 (± 9.3) years and non-chewers for 18.5 (± 12.9) years. Similarly, Khat chewers were significantly (P < 0.05) more likely to be drinking coffee, and to be adding salt in their coffee. On the other hand, alcohol drinking was significantly (P < 0.001) more common among non-chewers (30.9%) than Khat chewers (7.8%) (Table 2).

**Table 2:** Comparison of behavioral characteristics between Khat chewers and non-chewers in Butajira, Ethiopia.

	Chewers Freq (percent)	Non-chewers Freq (percent)	P value
**Current Smokers**	36 (10.8)	16 (4.8)	P < 0.05

**Current Coffee drinkers**	330 (98.8)	318 (96.4)	P < 0.05

**Adding salt in coffee**	281 (84.1)	238 (72.1)	P < 0.05

**Current Alcohol drinkers**	26 (7.8)	102 (30.9)	P < 0.001

Table 3 shows comparison of the physical measurements between Khat chewers and non-chewers. The mean (sd) diastolic blood pressure was significantly (P < 0.05) higher among Khat chewers 75.0 (11.6) mmHg than non-chewers, 72.9 (11.7) mmHg, P < 0.05. Similarly, the mean heart rate per minute was significantly higher among Khat chewers.

**Table 3:** Comparison of physical measurements and indices across Khat chewers and non-chewers in Butajira, Ethiopia.

Measurement	Mean (sd)		Mean difference	P value
			
	Chewers (n = 329)	Non Chewers (n = 319)		
Height (cm)	164.0 (9.2)	163.0 (9.3)	1.0	0.16

Weight (kg)	53.3 (9.1)	52.0 (9.4)	1.3	0.07

Body mass index (kg/m^2 ^)	19.8 (2.8)	19.5 (3.1)	0.3	0.26

Waist circumference (cm)	83.2 (8.5)	82.1 (7.8)	1.1	0.08

Hip circumference (cm)	93.4 (7.5)	92.5 (7.3)	0.9	0.11

Waist-to-hip ratio	0.89 (0.06)	0.89 (0.06)	0.0	0.63

Systolic blood pressure (mm Hg)	116.9 (17.8)	116.1 (16.8)	0.9	0.57

Diastolic blood pressure (mm Hg)	75.0 (11.6)	72.9 (11.7)	2.3	0.023

Heart rate (bpm)	76.3 (11.5)	73.9 (12.6)	2.4	0.012

Abbreviations: bpm = beats per minute; cm = centimeter; kg = kilogram; m^2 ^= square meter; mm = millimeter

Significantly more Khat chewers (30%) had sub-optimal diastolic blood pressure (DBP > 80 mmHg) than the non-chewers (21%); Chi-square = 7.65, P < 0.01. Whereas, 47% of Khat chewers and 45% on the non-chewers had sub-optimal systolic blood pressure (SBP > 120 mmHg). The difference in the proportion with sub-optimal systolic blood pressure was not significant (P > 0.05).

The prevalence of hypertension (SBP > = 140 mmHg or DBP > = 90 mmHg or reported use of antihypertensive drugs) was significantly higher among Khat chewers (13.4%) than non-chewers (10.7%), OR = 1.66 (95% CI 1.05, 3.13). Similarly, 30.9% of chewers and 28.5% of non-chewers were found to have pre-hypertension, which is a designation that helps to identify individuals at high risk of developing hypertension.

Multiple linear regression analysis was conducted in order to examine the independent effect of the behavioral characteristics (smoking, coffee and salt use, and alcohol drinking) that were found to vary significantly across Khat chewers and non-chewers. The analysis (Table 4) revealed that none of these behavioral characteristics accounted for a significant variability in diastolic blood pressure. Only sex and body mass index (BMI) accounted for significant variability in mean diastolic blood pressure among Khat chewers. [Sex: beta-coefficient = -5.87(95% CI -9.24, -2.49); BMI: beta-coefficient = 1.16(95% CI 0.71, 1.59)].

**Table 4:** Multiple linear regression analysis for determinants of diastolic blood pressure among Khat chewers in Butajira, Ethiopia.

	Beta coefficient	Standard error	95% Confidence interval for Beta coefficient
Sex (male, female)	-5.87	1.72	(-9.24, -2.49)

Age	0.07	0.08	(-0.09, 0.23)

Marital status (single, other)	2.64	7.89	(-12.88, 18.16)

Religion (Moslem, other)	-0.65	2.79	(-6.14, 4.84)

Ethnic Group (*Meskan*, other)	0.93	1.27	(-1.57, 3.43)

Literacy status (illiterate, literate)	2.74	1.50	(-0.21, 5.69)

Main occupation (farmer, other)	2.79	1.47	(-0.11, 5.68)

Body mass index	1.16	0.22	(0.71, 1.59)

Waist-to-hip ratio	-7.97	9.90	(-27.45, 11.50)

Smoking (yes, no)	-0.18	2.10	(-4.31, 3.95)

Coffee drinking (yes, no)	1.41	5.75	(-9.90, 12.72)

Alcohol drinking (yes, no)	-1.84	2.71	(-7.16, 3.48)

Adds salt in coffee (yes, no)	-0.03	1.75	(-3.47, 3.41)

## Discussion

This study compared mean systolic and diastolic blood pressure between adults in Butajira district who are Khat chewers and non-chewers. We demonstrated the association of Khat chewing with mean diastolic blood pressure, which suggests that Cathinone may have a more pronounced effect on diastolic blood pressure. Earlier studies have also suggested that chronic use of Khat predisposes to high blood pressure and other cardiovascular morbidity [6,20,27].

Our study suggests for the first time, using a comparative study design that long-term Khat chewing contributes to an isolated increase in diastolic blood pressure at the population level. The present finding confirms an earlier report of association between Khat chewing and elevated blood pressure from a cross sectional study in a similar population [6]. Our finding is also concurrent with other studies that reported the effect of Khat chewing on cardiovascular disease morbidity and mortality [5,8,19,20].

The potency of Khat leaves varies depending on freshness, origin or type, and the chewing pattern [12]. Amount of Khat chewed is difficult to estimate in quantitative terms. In this study we have not attempted to quantify the amount of Khat chewed. Instead, we used the duration and frequency of Khat use as a surrogate measure of level of exposure to Khat. The use of the "bundle" as the standard measure of the amount of Khat was not a viable option in the study area due to a huge variability in the size of the "bundle" observed in the market place, which results from the different varieties of the Khat plant and the way it was harvested and bundled, which in turn affect the proportion between stems, branches and leaves. This measure needs to be validated in the local context before it can serve as standard measure.

The concomitant use of other classical cardiovascular risk factors, such as smoking, alcohol, or dietary salt intake along with Khat may modify the extent of association between Khat chewing and blood pressure [6]. Reported alcohol drinking varied markedly between chewers and non-chewers, such that non-chewers, who were predominantly Christians, seem to be substituting Khat with alcohol. Our observation suggests a religious dichotomy in the use of the two substances in Butajira.

The ideal method of determining salt consumption at the population level would be through the measurement of 24 hour urinary sodium excretion. However, this was not possible in the present study largely due to logistics limitations. In the absence of such laboratory methods, a qualitative assessment of the salt use behavior may offer important clues on salt intake. However, as indicated in Table 4, our regression analysis revealed that salt use behaviors (such as adding salt in coffee) was not significantly associated with mean diastolic blood pressure. Thus, the significant differences in mean diastolic blood pressure and prevalence of sub-optimal diastolic blood pressure we demonstrated in this study are unlikely to be confounded by the behavioral characteristics.

Although previous studies commonly reported association between Khat chewing and alcohol consumption [10,28,29], our finding revealed a different picture, which is largely attributable to the predominantly Moslem religious background of our study population where alcohol consumption is prohibited.

Other studies [30,31] have reported that higher level of alcohol intake could increase blood pressure and the risk of coronary heart disease mortality. As non-chewers in our study population are more likely than the Khat chewers to report intake of alcohol, it is possible that alcohol intake has contributed to increased blood pressure among the non-chewers as well. Consequently, the difference in mean blood pressure between the two groups might have been narrowed down or diluted due to the effect of Khat chewing and alcohol consumption on blood pressure among chewers and non-chewers, respectively.

Our findings suggest sustained effects of regular Khat chewing on blood pressures, beyond the transient rise in blood pressure and heart rate that have been demonstrated in experimental studies [5,8,14,20]. Although the specific mechanism is yet to be understood, the selective effect on diastolic blood pressure in our study population may be explained by the peripheral vasoconstrictor effect of Cathinone [32], which may be sustained in regular Khat chewers.

The mean diastolic blood pressure and heart rate of Khat chewers in our study were significantly higher than that of non-chewers, (p < 0.05). On the other hand, the difference in mean SBP between the two groups was not statistically significant. The reason for a less marked effect of Khat chewing on systolic blood pressure in contrast to diastolic blood pressure needs further investigation.

Use of self reported age and Khat chewing data in a setting where record of vital statistics are not available, may be subject to recall bias. No mechanism was designed to countercheck the reported age or consumption behavior. The effect of other contributing factors and associated health problems such as Diabetes, kidney diseases, heart diseases, level of cholesterol etc, that require additional input was not assessed.

The mean diastolic blood pressure among Khat chewers was significantly higher than that of the non-chewers, by 2.1 mmHg. A blood pressure variation of 2.1 mmHg may appear insignificant at an individual level, but can be regarded as an important difference at a population level. It takes much effort to lower (shift to the left) the mean blood pressure distribution of a given population by 2.1 mmHg. Such reduction would also have a significant contribution to lowering the risk of morbidity and mortality related to cardiovascular diseases at the population level. It has been well established that *"a large number of people exposed to a small risk may generate many more cases than a small number exposed to high risk" *[33]. Population-based strategies that seek to shift the whole distribution of risk factors often have the potential to produce substantial reductions in disease burden [34].

The quantitative measurement of Cathinone and its metabolic products in the urine, as well as the measurement of 24-hour urinary excretion of Sodium was beyond the scope of the present study. It is suggested that future studies in the area of Khat chewing and cardiovascular health may allow a more quantitative analysis by conducting such measurements.

## Conclusions

Our study revealed that Khat chewing may contribute to elevated blood pressure, with a more pronounced effect on diastolic blood pressure. A considerably higher proportion of Khat chewers have sub-optimal DBP. The findings suggest that Cathinone might have a sustained effect as peripheral vasoconstrictor among regular Khat chewers.

The association of regular Khat chewing and elevated blood pressure levels in the population has significant relevance for public health. Programs for the prevention and control of high blood pressure in the population need to devise strategies to counter the expansion of Khat chewing and other substance use behaviors.

## Competing interests

The authors declare that they have no competing interests.

## Authors' contributions

WG, drafted the study proposal, developed survey instruments and collected and processed data. FT and TG conceived of the study, and participated in its design and coordination and helped to draft the manuscript. All authors participated in data analysis. WG drafted the manuscript. FT and TG reviewed and revised the manuscript, and approved the final form.

## Pre-publication history

The pre-publication history for this paper can be accessed here:

http://www.biomedcentral.com/1471-2458/10/390/prepub
